# Baicalin's Therapeutic Time Window of Neuroprotection during Transient Focal Cerebral Ischemia and Its Antioxidative Effects *In Vitro* and *In Vivo*

**DOI:** 10.1155/2013/120261

**Published:** 2013-06-25

**Authors:** Fafeng Cheng, Yi Lu, Xianggen Zhong, Wenting Song, Xueqian Wang, Xiaoguang Sun, Jianguo Qin, Shaoying Guo, Qingguo Wang

**Affiliations:** ^1^College of Basic Medicine, Beijing University of Chinese Medicine, Beijing 100029, China; ^2^Xiyuan Hospital of China Academy of Chinese Medical Sciences, Beijing 100091, China; ^3^Dongfang Hospital, The Second Clinical Medical College of Beijing University of Chinese Medicine, Beijing 100078, China; ^4^The Central Hospital of Handan, Handan, Hebei 056001, China

## Abstract

We investigated the effects of baicalin on an ischemia-reperfusion-induced brain injury model in rats and its antioxidative activities *in vitro* and *in vivo*. An ischemia-reperfusion injury of the brain via a middle cerebral artery occlusion (MCAO) was induced in rats. Baicalin was injected at different time points (0, 2, 4, and 6 h) after the MCAO was induced. Baicalin can improve neurological function and significantly decrease brain infarction within a time window of 4 h. Moreover, baicalin was able to reduce cell apoptosis and had the strong antioxidative effect of reducing reactive oxygen species production and malondialdehyde generation. In contrast, baicalin interfered with superoxide dismutase and nicotinamide adenine dinucleotide 2′-phosphate oxidase activities. Moreover, baicalin also exhibited strong neuroprotective effects against H_2_O_2_-mediated injury and improved the SOD activity of neurons. Furthermore, baicalin demonstrated good scavenging of hydroxyl radicals, superoxide anions, and DPPH radicals and exerted an additional effect of inhibiting xanthine oxidase. Baicalin showed beneficial effects against MCAO-induced injury within a 4 h time window, and its antioxidative effects both *in vitro* and *in vivo* may partly elucidate its mechanism of action.

## 1. Introduction

**Figure 1:**
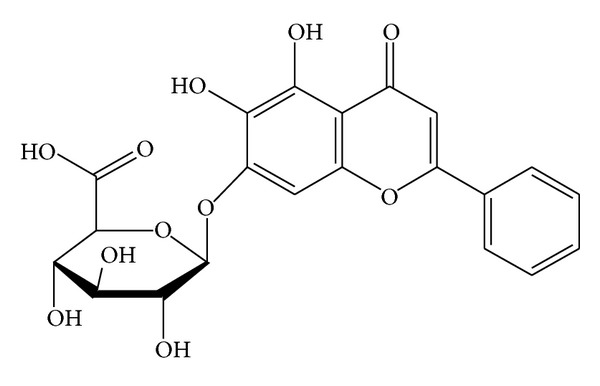
Chemical structure of baicalin.

Ischemic stroke is a life-threatening disease that is characterized by high morbidity and mortality. Although the pathogenesis of ischemic stroke remains unknown, several studies have suggested that free radicals might be involved in the inflammatory injury and oxidative stress that often accompanies this acute brain disease [1, 2]. Reactive oxygen species can attack proteins, deoxynucleic acid, and lipid membranes, thereby disrupting cellular function and integrity, leading to mitochondrial damage [3, 4]. These effects are commonly referred to as ‘‘oxidative stress” [5]. Therefore, removing free radicals or preventing their formation can be a potential therapeutic strategy. Several herbs in TCM have been used for thousands of years and were recently found to have antioxidative effects [6, 7]. Baicalin is one of several effective ingredients that is enriched in the dried root of the *Scutellaria baicalensis* Georgi, which is commonly used in traditional Chinese medicine (Figure 1).

Many studies have been conducted to investigate baicalin's health-promoting effects, in particular, its antibacterial, antivirus, anticancer, anti-inflammatory, and antioxidative effects [7–11]. Recent studies revealed that baicalin can protect against neuronal cell death and enhance neurological function following cerebral ischemia [7, 12, 13]. In addition, Zhang et al. [14] demonstrated that baicalin can pass through the blood-brain barrier and distribute within brain tissue, specifically in the hippocampus, striatum, cortex, and thalamus. 

In clinical practice, the timely treatment of stroke patients is not always possible, and the therapeutic time window is often correlated with therapeutic effect. In recent years, studies that focused on the therapeutic time window have attracted more attention from the committee of the Stroke Therapy Academic Industry Roundtable [15]. Although previous studies [12, 14, 16, 17] have investigated the therapeutic effects of baicalin, studies of its antioxidative activities and cytoprotective effects on primary neurons have been few, and the therapeutic time window of baicalin for cerebral ischemia has not yet been reported.

This study determined baicalin's scavenging activities of hydroxyl radicals, superoxide anions, and DPPH radicals and investigated its regulatory effects on the enzyme activities of such enzymes as xanthine oxidase, NADPH oxidase, and SOD. In this study, we focused on the therapeutic time window of baicalin for MCAO rats. Primary neurons were injured by H_2_O_2_ to test for neuroprotective effects after baicalin treatment. 

## 2. Materials and Methods

### 2.1. Animals and Drugs

Forty-eight healthy, male, C57BL/6 mice (25–30 g), 96 healthy, male, Sprague Dawley (SD) rats (230–250 g), and 10 newborn SD rats (born within 12 h) were purchased from Vital River Laboratories, Beijing, China (number SCXK (Beijing) 2008-0001) and housed in the Central Laboratory, Beijing University of Chinese Medicine, at a room temperature of 25 ± 1°C, with a relative humidity of 40–60%, automatic day-night rhythm and free access to standard lab chow and tap water. Any procedure involving animals and their care were performed according to the Prevention of Cruelty to Animals Act 1986 and the NIH Guidelines for Care and Use of Laboratory Animals and local laws.

Baicalin (5,6-dihydroxy-7-O-b-D-glucopyranosyl-2-penyl-4H-1-benzopyran-4-one) was purchased commercially from Sigma (Sigma Chemical Co., St. Louis, MO, USA). 

### 2.2. *In Vivo* Experiments

#### 2.2.1. MCAO Model

Transient focal ischemia was induced by a filament occlusion of the right middle cerebral artery [18]. Briefly, rats were anesthetized with 4% hydrochloride (350 mg/kg, i.p.) and kept under a heating lamp to maintain their core body temperature at 36.5 ± 0.5°C. The left common carotid artery, internal carotid artery (ICA), and external carotid artery (ECA) were surgically exposed under a dissecting microscope, and the ECA was coagulated at the bifurcation point. A 0.24 mm diameter nylon filament (tip diameter 0.32 ± 0.02 mm; Beijing Sunbio Biotech, Beijing, China) was inserted into the ICA through the ECA stump and was gently advanced (10 mm) to occlude the origin of the middle cerebral artery. After 1.5 h of MCAO, the filament was removed to restore blood flow (reperfusion) [19]. The rats were then allowed to recover after an incision closure and were housed individually. The MCAO procedure for the mice was similar to that for the rats, with the exception of the use of a nylon filament with a diameter of 0.16 mm and a round tip of 0.20 ± 0.01 mm.

#### 2.2.2. Groupings of Animals and the Administration of Drugs

**Figure 2:**
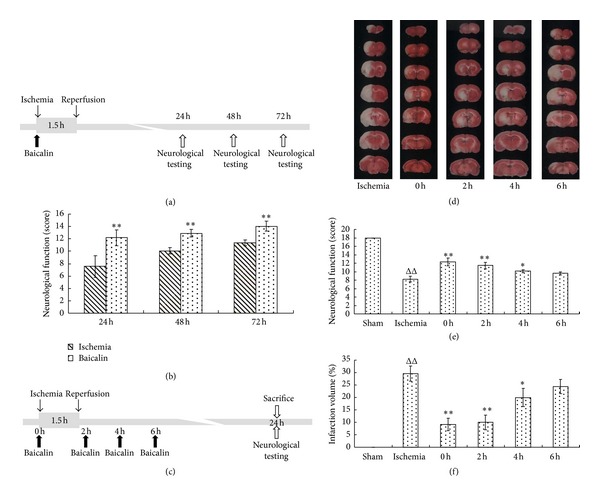
Therapeutic efficacy of baicalin in rats undergoing middle cerebral artery occlusion. (a) Flow chart of the determination of neurological function in injured rats at 24, 48, and 72 h after MCAO. (b) The neurological scores were determined in rats with MCAO (1.5 h) at various survival times after the onset of ischemia. Baicalin (15 mg/kg) or saline treatments were administered at 0 and 4 h after the onset of ischemia and after 24 h twice a day. The scores shown are presented as the means ± SEM. **P < 0.01, baicalin group is significantly different from the corresponding control group (*n* = 8 for each group). (c) A flow chart of the time window experiment of baicalin. (d) Seven coronal brain sections (2 mm thick) were selected for tetrazolium chloride staining. The red stain represents noninjured (normal) tissues; white represents the infarct region. (e) The infarct volume was quantified as a percentage of the total volume, with large infarcts corresponding to a more severe injury. (f) The neurological scores in rats after 1.5 h MCAO and 22.5 h reperfusion. Baicalin (15 mg/kg) treatments were first administered at 0, 2, 4, or 6 h after the onset of ischemia in the different groups, and saline was administered at 0 h in the ischemic group. In addition, 4 h after the first treatment, baicalin was administered again. ^ΔΔ^ *P* < 0.01, ischemic group versus sham; **P < 0.01,*P < 0.05, baicalin-treated groups versus ischemia group (*n* = 10 for each group).

Twenty-four SD rats were used to establish the MCAO model. After elimination of the animals that died or those in which the model was not performed properly, the animals were randomly assigned into two equally sized groups. Baicalin (15 mg/kg) [17] was injected via the tail vein at 0 and 4 h after the onset of ischemia and then twice daily from day 2. The MCAO with vehicle treatment group was injected with the same volume of saline (Figure 2(a)).

For the therapeutic time window experiments, 72 rats were randomly assigned to the sham, MCAO-only, and 0, 2, 4, and 6 h baicalin-treated groups. These animals were injected with 15 mg/kg baicalin (or 0.9 mL/100 g saline in the MCAO-only group). Baicalin treatments were first administered at 0, 2, 4 or 6 h after the onset of ischemia in the different groups, and saline was administered at 0 h in the MCAO-only group. All of the animals in baicalin treatment groups were given a second administration of baicalin 4 h after the first injection, and MCAO-only group received a second administration of saline 4 h after MCAO (Figure 2(c)). 

Mice were divided into sham, MCAO-only, and baicalin (15 mg/kg)-treated groups, with 16 animals in each group. All of the mice were subjected to a MCAO; those animals that died or those animals in which the MCAO failed were eliminated from the study. Baicalin (15 mg/kg) or saline was administered via the tail vein at 0 and 4 h after the onset of ischemia. After 24 h, 5 mice from each group were sacrificed, and their brains were prepared for the TUNEL and ROS detection assays. The brain homogenates from the other animals in each group were collected to determine the NADPH oxidase, SOD, and MDA levels.

#### 2.2.3. Neurological Evaluation

The neurological function of the surviving rats was evaluated with a scoring system that was previously described by Garcia et al. [20] which consists of 6 tests: spontaneous activity, symmetry in the movement of their 4 limbs, forepaw outstretching, climbing, body proprioception, and response to vibrissae touch. The score obtained from each rat was the sum of the 6 test scores with the maximum score of 18. The neurological evaluation was performed at 24 h after the onset of ischemia for rats in the time window experiments and at 3 other time points (at 24, 48, and 72 h after MCAO) for the other two groups (Figures 2(a) and 2(c)). 

#### 2.2.4. Calculation of the Infarct Volume

After the evaluation for neurological function, the rats were sacrificed and their brains were harvested for TTC staining (Nanjing Greensynthesis Biochemical Co., Ltd., Nanjing, Jiangsu, China). The percentage of the infarct volume from the total brain volume represented the degree of cerebral infarction. Serial coronal sections (2 mm thickness) were prepared and soaked in 2% TTC phosphate buffer at 37°C for 10 minutes in the dark. In this assay, the normal brain tissues were stained red, while the infarct tissues remained unstained (white). The sections were soaked in 4% paraformaldehyde phosphate buffer for 30 minutes, arranged serially, and then scanned (Tsinghua Unisplendour A688, Xi'an, China). The red and white stained areas were measured using a computer color multimedia image analysis system (Image-Pro Plus6.0, Media Cybernetics, WY, USA). The percentage of infarction was determined by the following equation [18].

(1) $$\begin{array}{c} \%\text{Infarction volume}=\left(\frac{\text{Infarction volume}}{\text{Total volume of slice}}\times 100\right). \end{array}$$

#### 2.2.5. ROS Measurement through DHE Staining

Brain ROS production was determined using dihydroethidium (DHE) microfluorography [18]. DHE is a cell permeable dye, which can be oxidized into ethidium and other products by superoxide [21]. The animals were sacrificed after 24 h MCAO, and the brains were removed, frozen, and then cryosectioned at a thickness of 20 *μ*M. Sections obtained from the prefrontal cortex were collected and an ROS fluorescence detection kit (Genmed, WY, USA) was used. The DHE solution was superfused on to the brain sections for 60 minutes, and fluorescence intensity was detected using fluorescence microscopy (ZEISS, LSM510 meta, Germany). The fluorescence intensity of 5 different fields in the prefrontal cortex of the ischemic hemisphere was averaged and expressed as relative fluorescence units (RFU) [22]. 

#### 2.2.6. TUNEL Detection

After sampling and cutting the brain sections as previously described, TUNEL staining was performed using a detection kit for programmed cell death (In Situ Cell Death Detection Kit, TMR Red, Roche, USA) according to the manufacturer's instruction. Five areas obtained from each section were examined by fluorescence microscopy (ZEISS, LSM510 meta, Germany) in the prefrontal cortex of the ischemic hemisphere, and the TUNEL-positive cells were quantified [23].

#### 2.2.7. Measurement of NADPH Oxidase

The forebrain from the ischemic side was homogenized with saline for the measurement of NADPH oxidase activity. The cytosolic (supernatant) and membrane (pellet) fractions were separated using a Protein Extraction Kit (Transmembrane Protein Extraction Kit, Novagen, Darmstadt, Germany). The membrane fraction was then used for further analysis of NADPH oxidase enzymatic activity [24] and performed according to previously described protocols [24, 25]. Aliquots of the brain homogenate were incubated with NADPH at 37°C, and the NADPH oxidase enzymatic activity was determined every 10 minutes by measuring the reduction of NADPH with a plate reader spectrophotometer (450 nm) and a Radical Detector kit (Genmed, USA). The NADPH oxidase activity was normalized by the amount of protein in each sample, and the increase in absorbance was measured every 10 and 20 minutes. The activity was calculated as mU/(g prot × min).

#### 2.2.8. Detection of SOD and MDA

The brain was removed 24 h after ischemia, and the cortex was isolated from the lesioned hemisphere and homogenized, and the supernatant was collected [18]. The MDA production reflects the degree of lipid peroxidation injury [26]. The MDA was determined using a kit according to the manufacturer's directions (Nanjing Jiancheng Bioengineering Institute). In addition, the SOD activity was determined using the xanthine oxidase method [26] according to the manufacturer's directions (Nanjing Jiancheng Bioengineering Institute).

### 2.3. *In Vitro* Experiments

#### 2.3.1. Protection against H_**2**_O_**2**_-Induced Cell Toxicity in Primary Rat Cortical Neuronal Cultures

Primary rat cortical neuronal cultures were prepared from the cerebral cortices of newborn SD rats. Twelve-day-old cultures were used in this study. The cells were seeded in a 96-well flat bottom plate at a density of 2.0 × 10^5^ cells/mL and were either incubated with different concentrations of H_2_O_2_ or treated with baicalin at a concentration of 10, 20, 40, 80, and 200 *μ*M in addition to 300 *μ*M H_2_O_2_. After 16 h of incubation [27], the cell viability and toxicity were determined. The cell viability was assessed by a colorimetric assay using 3-(4,5-dimethylthiazol-2-yl)-2,5-dip-henyltetrazolium bromide (MTT, Sigma Chemical Co., St. Louis, MO, USA), which yields a blue formazan product in living cells, but not in dead cells or their lytic debris. MTT was dissolved in a serum-free glucose solution and added to the culture at a final concentration of 0.5 mg/mL. After 2 h of incubation at 37°C, the medium was carefully removed, and 100 *μ*L DMSO was added to each well. The plates were then read at 580 nm on a spectrophotometer (Bio-Rad, CA, USA) and the results of the neuronal viability was determined as a percentage compared to controls [27]. The LDH release in the medium was measured using a kit from Nanjing Jiancheng Bioengineering Institute and performed according to the manufacturer's directions. The results were expressed as U/L, and the SOD activity in the neurons was also detected. The cells were washed with ice cold PBS, collected in 0.1 M PBS/0.05 mM EDTA buffered solution, and then homogenized using sonication. The homogenates were centrifuged at 10000 r/min for 10 min at 4°C, and the SOD activity in the supernatants was determined using a kit from Nanjing Jiancheng Bioengineering Institute and performed according to the manufacturer's directions.

#### 2.3.2. Detection of Antioxidative Activity of Baicalin in Several Chemical Systems

*Assay of Hydroxyl Radical Scavenging.* The hydroxyl radicals were generated in an H_2_O_2_-FeSO_4_ system by the oxidation of FeSO_4_ and were assayed by a colorimetric change of salicylic acid, according to previously described protocols [28]. In this experiment, the hydroxyl radicals were generated in a reactive solution containing 0.5 mL of 9 mM FeSO_4_, 0.5 mL of 9 mM salicylic acid-ethanol solution, and the samples were to be tested at different concentrations. Lastly, 5 mL of 9 mM H_2_O_2_ was added to this reactive solution. The mixture was incubated at 37°C for 1 h and the absorbance of the hydroxylated salicylate complex was measured at 510 nm. The inhibition of hydroxyl radical production was calculated as follows: inhibition rate (%) = (the absorbance of the control group − the absorbance of the test group)/the absorbance of the control group × 100%.

*Superoxide Anion Scavenging Effect.* Superoxide anions were generated enzymatically by the xanthine/xanthine oxidase system according to Toda et al. [29]. Briefly, the reaction mixture consisted of 25 mL of 40 mM sodium carbonate buffer (pH 10) containing 0.1 mM EDTA (pH 10.0), 0.06 mL of 10 mM xanthine, 0.03 mL of 0.5% bovine serum albumin, 0.03 mL of 2.5 mM nitroblue tetrazolium, and 0.06 mL of baicalin (dissolved in DMSO). Baicalin was dissolved in 0.2% DMSO to give final concentrations of 400, 300, 100, 80, 40, 20, and 10 *μ*M. The reaction was initiated by the addition of 0.12 mL xanthine oxidase (0.04 units) into each tube, and the absorbance at 560 nm was recorded for 90 s (by the formation of blue formazan). The control experiments were performed by replacing the sample solution with the same volume of 0.2% DMSO. 

*DPPH Radical Scavenging Activity.* The radical scavenging activity on DPPH was detected according to previously described protocols [28]. First, 1 mL of 100 mM acetate buffer (pH 5.5), 1.87 mL of ethanol, and 0.1 mL of ethanol solution of 3 mM of DPPH were mixed in a test tube. Then, 0.03 mL baicalin (dissolved in DMSO) was added to the test tube and incubated at 25°C for 20 min. The absorbance at 517 nm (DPPH, *ε* = 8.32 × 10^3^) was recorded. As a control, 0.03 mL of DMSO was added to the control tube. The scavenging activity was calculated from a decrease in the absorbance and expressed as follows: scavenging activity (%) = (*A* − *B*)/*A* × 100%, where “*A*” represents the absorbance of the control tube and “*B*” represents the absorbance of the experimental tube.

*Inhibition of Xanthine Oxidase Activity.* The reaction mixture consisted of 2.5 mL of 40 mM sodium carbonate buffer (pH 10) containing 0.04 mL of 0.1 mM EDTA, 0.04 mL of 10 mM xanthine, and 0.04 mL sample solution (dissolved in 0.2% DMSO). The reaction was initiated by the addition of 0.01 mL of xanthine oxidase (0.04 units), and the absorbance at 293 nm was recorded for 90 s. The control experiments were performed as described previuosly [30].

*Total Antioxidant Capacity.* The total antioxidative capacity was determined using a spectrophotometric assay kit (Nanjing Jiancheng Bioengineering Institute) and performed according to the manufacturer's instructions. In brief, 30 *µ*L baicalin at different concentrations was added to the reaction buffer containing xanthine, xanthine oxidase, and hydroxylamine. After a 40 min incubation at 37°C, the accumulation of nitrite was quantified by the Griess reaction. The antioxidative capacity is inversely correlated with the concentration of nitrate [31]. These results were normalized according to the manufacturer's instructions and expressed as U/L.

### 2.4. Statistical Analysis

The data were analyzed using SPSS 16.0 software. A one-way analysis of variance was performed and was followed by a post hoc analysis for significance using the Student-Newman-Keuls multiple comparison test. All of the values are expressed as the mean ± SEM. A value of *P* < 0.05 was considered to be statistically significant.

## 3. Results

### 3.1. Baicalin Effects on MCAO-Induced Cerebral Damage

Simultaneous administration of baicalin (15 mg/kg) with the onset of ischemia could significantly improve the neurological function of rats at 24, 48, and 72 h after MCAO (Figure 2(a)). Rats in both the MCAO-only and baicalin-treated groups showed a trend towards neurological functional recovery (Figure 2(b)). Importantly, animals in the baicalin-treated group showed enhanced neurological function compared with the MCAO-only group at each time point. We chose the 24 h time point after MCAO to assess the neurological function and infarct volume in our time window experiments (Figure 2(c)). We found that administration of baicalin at 0, 2, and 4 h after MCAO can reduce the infarct size by 68.94%, 66.3%, and 32.6%, respectively (Figures 2(d) and 2(e)), and significantly increased the rats' neurological scores (Figure 2(f)) compared with the MCAO-only group. 

**Figure 3:**
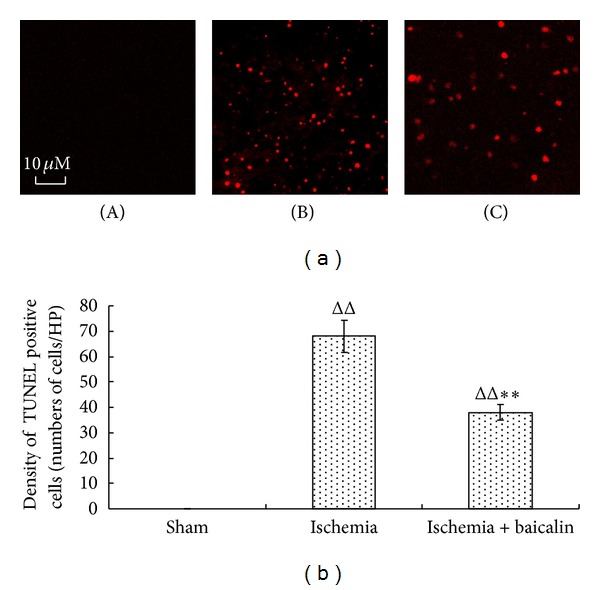
Effects of baicalin on TUNEL staining in the prefrontal cortex of MCAO mice. After 24 hr of MCAO, apoptotic cells ((a), ×400) were detected in regions of the prefrontal cortex. The apoptotic cells were labeled with red fluorescence, (A) sham group, (B) ischemia group, and (C) baicalin-treated group. Five animals were selected from each group and 3 sections were selected from each site. Five 400-fold fields of view were randomly selected from each section to quantify the mean of the positive cells. The results are expressed as the mean ± SEM (b). ^ΔΔ^ *P* < 0.01, ischemia group versus sham; **P < 0.01, baicalin-treated group versus ischemia group.

No significant differences were observed between the groups treated with baicalin at 6 h after MCAO and the MCAO-only group. Thus, baicalin decreased the infarct volume and improved neurological function in a time window of 4 h. Cellular apoptosis is an important mechanism of neuronal injury after brain ischemia. The terminal deoxynucleotidyl transferase dUTP nick end labeling (TUNEL) method was used to test the neuroprotective effects of baicalin in MCAO rats [23]. A large number of TUNEL-positive neurons were observed in the prefrontal cortex 24 h after ischemia (Figure 3(a)). In the baicalin treated group (15 mg/kg), the number of TUNEL-positive neurons was reduced by 44% in the prefrontal cortex compared with the MCAO-only group (Figure 3(b)).

### 3.2. The Antioxidative Effects of Baicalin in MCAO Mice

**Figure 4:**
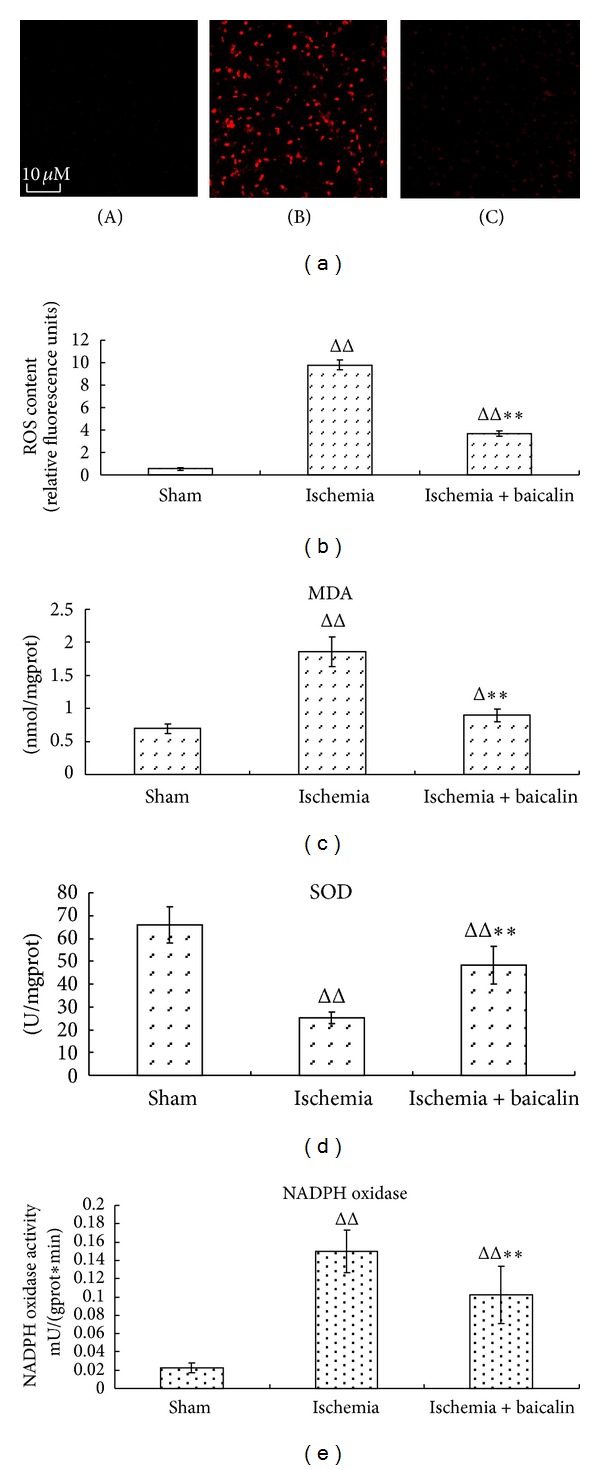
Effects of baicalin on the oxidative stress in mice undergoing a middle cerebral artery occlusion. Twenty-four hours after the onset of MCAO, the ROS were detected in regions of the prefrontal cortex using DHE staining. The relative intensity of the red fluorescence represents the ROS content in the prefrontal cortex region of the injured hemisphere in the prefrontal cortex, (A) sham group, (B) ischemia group, and (C) baicalin treatment group ((a), DHE staining ×200). The relative fluorescence intensity in 5 sites obtained from one section was determined by fluorescence microscopy. The mean value of the ROS content was calculated and expressed as the mean ± SEM, *n* = 5 (b). The MDA content, NADPH oxidase activity, and SOD activity are shown in (c), (d), and (e), respectively (*n* = 7). ^ΔΔ^ *P* < 0.01, ischemia group versus sham; **P < 0.01, baicalin-treated group versus ischemia group.

Reactive oxygen species (ROS) production was detected using DHE staining. As shown in Figure 4(a), no fluorescence was detected in the brains obtained from the sham mice (Figure 4(a)). A large number of neuronal cells contributed to the significantly enhanced fluorescence observed in the prefrontal cortex region at 24 h after MCAO. After treatment with baicalin, the number of fluorescent neuronal cells was reduced, and the fluorescence intensity was decreased. A quantitative analysis showed that the fluorescence in the cortex was significantly decreased in the baicalin group compared with the MCAO-only group (*P* < 0.01, Figure 4(b)). In another assay, the malondialdehyde (MDA) content in the brain tissue was substantially increased (approximately three times) compared with the sham group 24 h after MCAO, and the MDA content was more than 50% less in the MCAO-only group compared to the baicalin-treated group (Figure 4(c)). These results are consistent with our ROS detection data. The antioxidative effects of baicalin were associated with the regulation of the activity of superoxide dismutase (SOD) and nicotinamide adenine dinucleotide 2′-phosphate (NADPH) oxidase. SOD, an endogenous antioxidase, is constitutively active in normal brain tissues [32]; however, in this study, its activity was significantly decreased 24 h after MCAO, which is consistent with the result obtained by a previous study [33]. Interestingly, we found that the SOD activity in the baicalin group was approximately twice that of the MCAO-only group (Figure 4(d)). NADPH oxidase plays an important role in ROS generation after cerebral ischemia [34, 35], and its activity is normally low in uninjured animal brain tissues but significantly increases after cerebral ischemia. As shown in Figure 4(e), baicalin significantly inhibited the increase of NADPH oxidase. These results suggest that baicalin has strong antioxidative effects on reducing ROS production and MDA generation, as well as regulates SOD activity and NADPH oxidase activity after MCAO-induced injury. 

### 3.3. Neuroprotective Effects on H_**2**_O_**2**_-Induced Neuronal Injury

**Figure 5:**
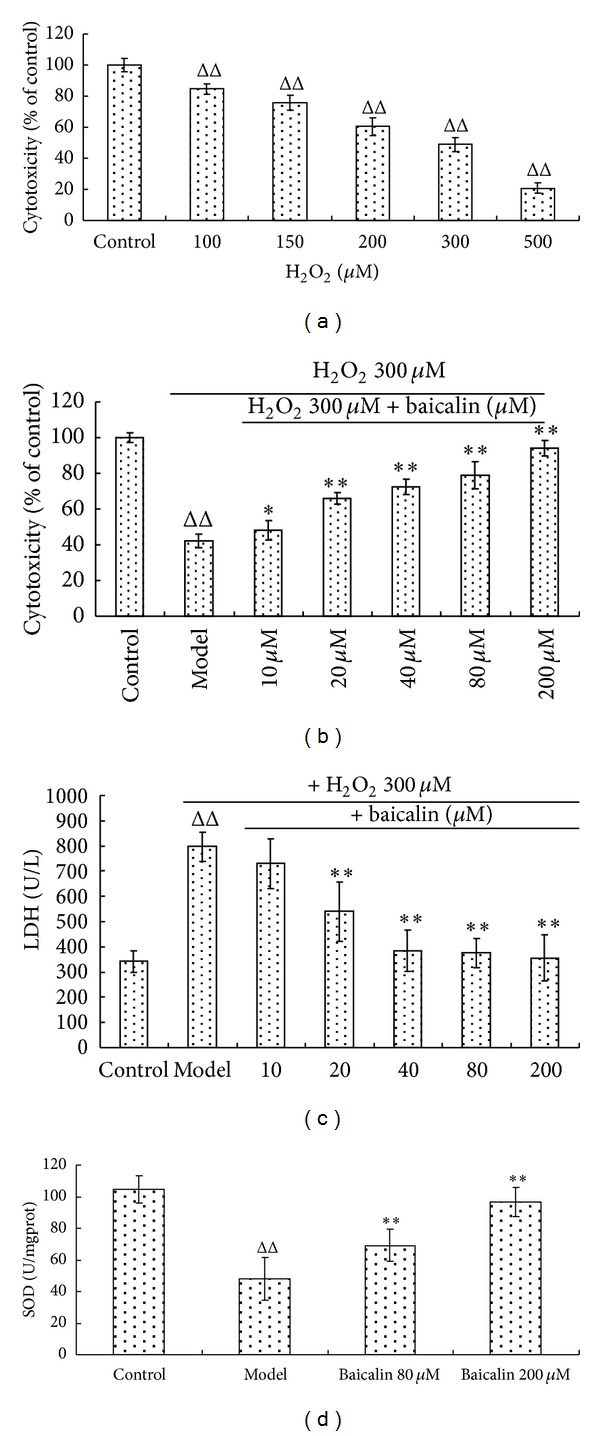
Protection effects of baicalin against H_2_O_2_-induced cell toxicity in primary rat cortical neuronal cultures. (a) The cell viability of primary rat cortical neuronal cultures injured by different concentrations of H_2_O_2_ after incubation for 16 h. An H_2_O_2_ concentration of 300 *μ*M was chosen for the following experiments. (b) The effects of baicalin at concentrations of 10, 20, 40, 80, and 200 *μ*M on the viability of H_2_O_2_ (300 *μ*M) injured neurons. (c) Baicalin reduced the levels of LDH in the cell supernatant. (d) Baicalin at 80 and 200 *μ*M increased the SOD activity of neuronal cultures. ^ΔΔ^ *P* < 0.01, H_2_O_2_-treated groups versus control; **P < 0.01,*P < 0.05, baicalin-treated groups versus H_2_O_2_-only (*n* = 8 for each group).

After the primary rat cortical neuronal cultures were incubated with different concentrations of H_2_O_2_ for 16 h, we found that cell viability decreased with increasing H_2_O_2_ concentrations. Treatment with 300 *μ*M H_2_O_2_ resulted in only 45% cell survival compared with the untreated group. (Figure 5(a)). This *in vitro* model was used to test the antioxidative effects of baicalin. MTT test results showed that with increasing concentrations of baicalin, there was a corresponding increase in cell survival, demonstrating a dose-dependent relationship between baicalin and cell viability (Figure 5(b)). We found no significant differences in the cell viability between the 200 *μ*M baicalin-treated and normal groups. Accordingly, the LDH leakage in the H_2_O_2_-only group was much higher than in the untreated cells, and this leakage decreased with the addition of baicalin, as shown in Figure 4(c). Baicalin (200 *μ*M) decreased LDH leakage by more than 50% compared with the H_2_O_2_-only group, which is not significantly different from untreated cells (Figure 5(c)). These results indicate that baicalin exhibited strong neuroprotective effects against H_2_O_2_-mediated injury. Furthermore, baicalin can also significantly improve the SOD activity of neurons, the activity of which decreased after H_2_O_2_-induced injury (Figure 5(d)). 

### 3.4. Oxygen Radical Scavenging and Other Baicalin Antioxidative Activities

**Figure 6:**
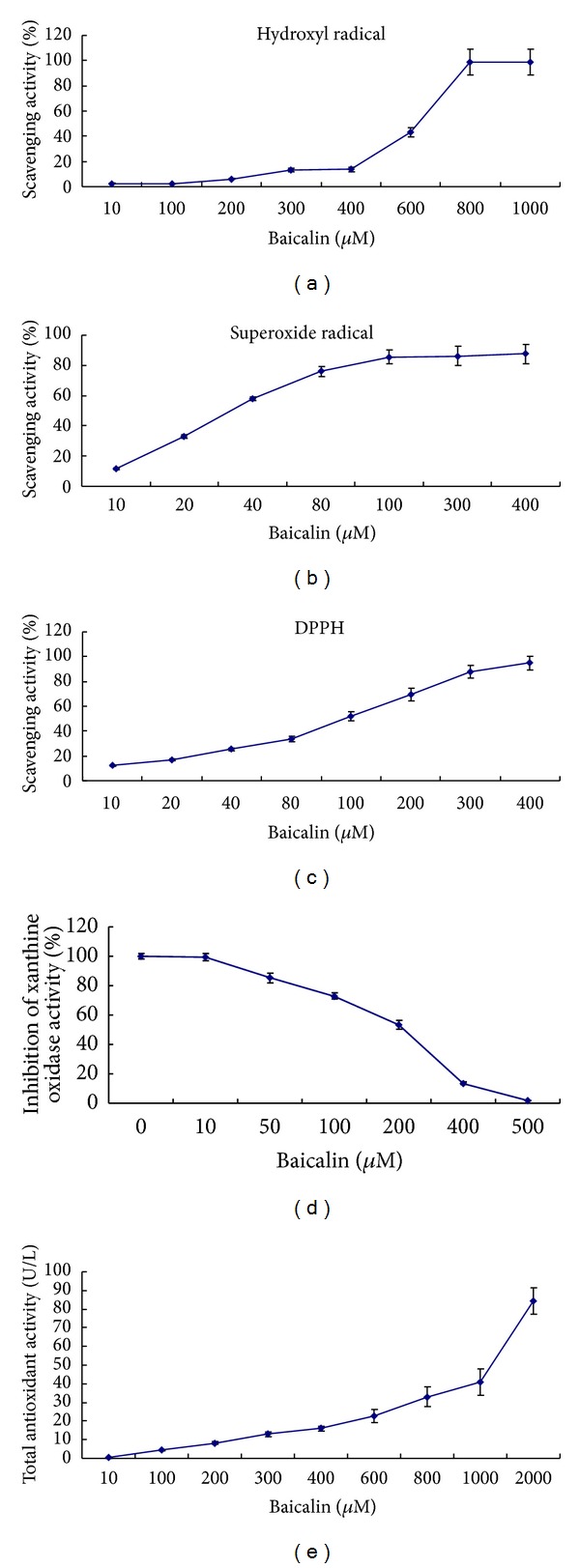
Free radical scavenging and antioxidative activity of baicalin. (a) Hydroxyl radical scavenging activity of baicalin. (b) Superoxide anion scavenging effect. (c) DPPH radical scavenging activity. (d) Inhibition of xanthine oxidase activity. (e) Total antioxidative activity. All of the experiments were repeated 3 times.

Several tests were performed to investigate the antioxidative activities of baicalin in various signaling systems. First, the antioxidative activity of baicalin was determined by measuring the scavenging of hydroxyl radicals (OH^−^). The hydroxyl radical is the most reactive member of the reactive oxygen species, and it induces severe damage to DNA, lipids, and proteins. The OH^−^ scavenging activity of baicalin is assessed by its competition with salicylic acid for OH^−^ radicals in the OH^−^ generating/detecting system. In this study, the hydroxyl radical-scavenging effect of baicalin was found to be 43.36% and 98.71% at concentrations of 600 *µ*M and 1000 *μ*M, respectively (Figure 6(a)). The IC_50_ value was found to be 390 *μ*M. Thus, baicalin is a good scavenger of hydroxyl radicals. The scavenging activity of baicalin against the enzymatically generated superoxide anions in the xanthine/xanthine oxidase pathway is shown in Figure 6(b). Baicalin reduced superoxide anion formation in a dose-dependent manner and with an IC_50_ value of 134.66 *μ*M. Next, the decolorizing activity of DPPH was detected. We found that the DPPH radical scavenging activity increased with increasing concentrations of baicalin (Figure 6(c)). This result indicated that baicalin can quench the DPPH radical with an IC_50_ value of 64.92 *μ*M. 

The antioxidative activities of some flavonoids (e.g., quercetin and luteolin) are caused by both radical scavenging and the inhibition of enzymatic activity. Xanthine oxidase is known to convert hypoxanthine to xanthine and finally to uric acid. To evaluate the effects of baicalin on xanthine oxidase activity, the formation of uric acid was measured. Baicalin inhibited the oxygen-atom-transfer reaction in a dose-dependent manner (Figure 6(d)). Moreover, the IC_50_ value was calculated as 134.66 *μ*M. Finally, the total antioxidative capacity was assessed. Consistent with the above results, baicalin showed a strong total antioxidative capacity and exhibited dose-dependent effects (Figure 6(e)). 

In conclusion, baicalin showed good scavenging activity on hydroxyl radicals, superoxide anions, and DPPH radicals and exerted the additional effect of inhibiting xanthine oxidase.

## 4. Discussion

This study defined a therapeutic time window for the neuroprotective effects of baicalin in MCAO-induced ischemic rats. Furthermore, our results provided important insight into the antioxidative effects of baicalin *in vitro* and *in vivo*. 

There is accumulating evidence that *Scutellaria baicalensis* Georgi containing various flavonoids including baicalin possess neuroprotective effects against cerebral ischemic injury [16, 36]. Previous studies reported that baicalin exhibited protective effects in a model of focal cerebral ischemia [12, 16]. However, its therapeutic time window was unclear. Abundant evidence has shown that a number of neuroprotective drugs, which had shown efficacy in ischemic animal models in previous studies, have failed in clinical trials due to inadequate investigation of optimal therapeutic timewindows [18]. Therefore, it is necessary to explore therapeutic time windows for drug treatment in animal models as a part of the evaluation of the neuroprotective effects of these drugs during ischemia. In this study, we confirmed that baicalin significantly improved the neurological score and reduced the infarct volume. Furthermore, the therapeutic time window of baicalin was established at 4 h in MCAO injured rats. Time window is crucial in clinical ischemia treatment. Stroke patients generally get medicine treatment after certain time interval since ischemia onset; so, it is very important to have a certain time window for proposal medicine. For instance, time window of thrombolytic drugs is 3 to 6 h. With a longer time window, more chances will provide to protect patients. In this study, mice experiments showed that the time window of baicalin is 4 h, which indicates a promising clinical use. 

Additional lines of evidence from various studies have converged to suggest that oxidative stress, characterized by an overproduction of ROS, contributes to ischemia-induced injury in the following ways: lipid peroxidation; protein denaturation; inactivation of enzymes; nucleic acid and DNA damage, which results in the release of Ca^2+^ from intracellular stores; damage to the cytoskeletal structure; and chemotaxis [1, 2, 37]. To explore a possible mechanism of baicalin on the relief of oxidative stress induced by ischemia-reperfusion injury, we investigated the baicalin-dependent effects on ROS generation, MDA content, SOD activity, and NADPH oxidase activity in MCAO mouse brain tissue homogenates. Our results showed that baicalin significantly reduced ROS production and decreased MDA content, indicating that baicalin attenuates cerebral ischemia-reperfusion-induced oxidation injury *in vivo*.

An H_2_O_2_-induced primary neuronal injury *in vitro* model was employed to test the antioxidative and neuroprotective effects of baicalin. Cortical neurons are particularly vulnerable to H_2_O_2_ injury because of their relatively low levels of antioxidant enzymes and dependence on mitochondrial respiration [38]. After incubation with 300 *μ*M H_2_O_2_, the neurons exhibited a decrease in SOD activity, an increase in LDH leakage, and 55% cell death. Treatment with baicalin significantly increased the cell viability, reduced the LDH leakage, and enhanced the SOD activity. These results indicate that baicalin exerts beneficial antioxidative effects. 

ROS such as superoxide anions (O^2−^), peroxynitrite, and hydroxyl radicals have significant cellular effects that lead to tissue destruction and cell death. These effects are a consequence of high concentrations of ROS that exceed the ability of antioxidant defense mechanisms to counterbalance the damaging effects. ROS are a natural by-product of oxygen metabolism. O^2−^ is the primary ROS, and it can transform into other ROS [37]. Moreover, O^2−^ is produced in tissues via a number of enzymatic reactions and common cellular sources, including mitochondrial respiration, xanthine oxidase, NADPH oxidase, and nitric oxidase synthetase (NOS) [39–42]. In our study, several tests were performed *in vitro* and *in vivo* to determine the antioxidative mechanisms of baicalin. 

SOD can catalyze the dismutation of superoxide into oxygen and hydrogen peroxide, which is important in antioxidant defense *in vivo *[32, 33]. To detect the antioxidative activity, it is important to assess this enzyme [43]. After the ischemia-reperfusion injury, the SOD activity in brain tissue was decreased. Baicalin treatment increased the SOD content, ameliorating the severity of ischemic stroke. The results obtained from the SOD detection in neuronal cultures were consistent with the tests performed *in vivo*. Both sets of results verified the upregulation effects observed with baicalin treatment on SOD activity. Recent evidence indicates that NADPH oxidase plays a key role in cerebral ischemia [34, 35], and some NADPH oxidase regulators have been previously shown to be cerebroprotective [44]. This study demonstrated that baicalin negatively regulates the NADPH oxidase activity that was increased during the cerebral ischemia-reperfusion injury.

To investigate baicalin-dependent effects in various signaling pathways, we measured the scavenging activities of hydroxyl radicals, superoxide anions, and DPPH radicals and the inhibition of xanthine oxidase. Our results showed that baicalin demonstrated beneficial effects on both direct free radical scavenging activities and the inhibition of xanthine oxidase. We concluded that baicalin has beneficial antioxidative effects through a variety of mechanisms. Baicalin, a flavonoid antioxidant, plays multiple roles including the direct quenching of reactive oxygen species, the inhibition of enzymes involved in the production of the ROS (e.g., xanthine and NADPH oxidases), the increase of enzymatic activity involved in antioxidant defenses (e.g., SOD), (potentially) the chelation of low valent metal ions (i.e., Fe^2+^ or Cu^2+^) [30, 45]. 

In summary, baicalin showed beneficial effects against MCAO-induced injury with a therapeutic time window of 4 h after occlusion. Its antioxidative effects both *in vitro* and *in vivo* might partly elucidate its mechanism of action. Further investigation is necessary to characterize the neuroprotective effects of baicalin, which may provide insight into novel therapeutic strategies for ischemia and other neuronal injuries.

## 5. Conclusion

Baicalin has neuroprotective effects in MCAO rats within a therapeutic time window of 4 h. Baicalin showed good antioxidative effects both *in vitro* and *in vivo*. Baicalin can play multiple roles including oxygen radical scavenging and regulation of enzymes.

## Abbreviations

MCAO: Middle cerebral artery occlusion
ROS: Reactive oxygen species
SOD: Superoxide dismutase
MDA: Malondialdehyde
NADPH: Nicotinamide adenine dinucleotide 2′-phosphate.
